# Parkinson's Disease Detection Using Isosurfaces-Based Features and Convolutional Neural Networks

**DOI:** 10.3389/fninf.2019.00048

**Published:** 2019-07-02

**Authors:** Andrés Ortiz, Jorge Munilla, Manuel Martínez-Ibañez, Juan M. Górriz, Javier Ramírez, Diego Salas-Gonzalez

**Affiliations:** ^1^Department of Communications Engineering, Universidad de Málaga, Malaga, Spain; ^2^Department of Signal Theory, Networking and Communications, University of Granada, Granada, Spain

**Keywords:** deep learning, isosurfaces, Parkinson's disease, convolutional neural networks, computer-aided diagnosis

## Abstract

Computer aided diagnosis systems based on brain imaging are an important tool to assist in the diagnosis of Parkinson's disease, whose ultimate goal is the detection by automatic recognizing of patterns that characterize the disease. In recent times Convolutional Neural Networks (CNN) have proved to be amazingly useful for that task. The drawback, however, is that 3D brain images contain a huge amount of information that leads to complex CNN architectures. When these architectures become too complex, classification performances often degrades because the limitations of the training algorithm and overfitting. Thus, this paper proposes the use of isosurfaces as a way to reduce such amount of data while keeping the most relevant information. These isosurfaces are then used to implement a classification system which uses two of the most well-known CNN architectures, LeNet and AlexNet, to classify DaTScan images with an average accuracy of 95.1% and AUC = 97%, obtaining comparable (slightly better) values to those obtained for most of the recently proposed systems. It can be concluded therefore that the computation of isosurfaces reduces the complexity of the inputs significantly, resulting in high classification accuracies with reduced computational burden.

## 1. Introduction

Parkinson's Disease (PD) is a progressive and chronic neurodegenerative disorder of the central nervous system that affects movement. PD increases its occurrence with age and, currently, has a prevalence between 1 and 3% in the population over 65 years of age, becoming the second most common neurodegenerative disorder after the Alzheimer's disease. The origin of the disease has been not determined yet but it is related to the loss of dopaminergic neurons, which causes reduced quantities of dopamine transporters in the striatum (Simuni and Rajesh, 2009). In fact, dopaminergic neurons produce dopamine, a neurotransmitter, in the substantia nigra and and it is transported to the striatum, composed by caudate and putamen, through the nigrostriatal pathway.

To date there is no cure for PD but early diagnosis allows limiting the rate of progression by applying effective management and may help to develop new therapeutic methods. Diagnosis of PD is usually based on clinical examinations that analyze different motor symptoms such as tremor, bradykinesia, rigidity and postural instability (Eckert et al., 2007), along with the response to levadopa. Levadopa is a chemical product that converts to dopamine so that PD is confirmed whether symptoms reduce after levadopa is administered during a period of time. However, PD can be confused with other parkinsonian syndromes and in the early stages of the disease symptoms are still mild and the response to levadopa are not so clear, which may result in difficult diagnosis. As a consequence, functional neuroimaging are then usually used to improve the early diagnosis of the disease.

Single Photon Emission Tomography (SPECT) using the ^123^*I* − *ioflupane* radiotracer (also known by its tradename DaTSCAN) is commonly used for diagnosis of PD. DaTSCAN binds to the dopaminergic transporters at the striatum, allowing to measure quantitatively the amount of DaTSCAN in this region. DaT SPECT or DaTSCAN imaging results in multiple grayscale images captured by a gamma camera rotated through 360° around the body where the intensity of each pixel is directly correlated with the presence of radiotracers registered by the gamma camera. These 2D projections are then reconstructed to produce a 3D image. Comparing to healthy individuals, the resulting image for PD patients displays lower intensity and/or asymmetry in the striatal region. This way, DaTSCAN can be used to differentially diagnose PD with respect to normal or other diseases presenting similar symptons (NC) by detecting dopaminergic deficits.

In recent years, different works have analyzed DaTSCAN images for use in the clinic as an aid to visual reporting. Thus, a range of of semi-quantification methods can be found in the literature (Taylor and Fenner, 2017). These methods compute SBRs (Striatal Binding Ratios) from both, with and without consideration of the caudates, using different methods and establishing certain limits and likelihood of disease being present. The clinician must eventually interpret the results to come to an overall decision. At this point, machine learning algorithms can be used to help with such decision. Machine learning algorithms can combine multiple input variables describing different features to produce a single value that helps the clinician. These methods search statistical differences between two groups, PD and control (NC), using statistical learning (Rojas et al., 2013; Martínez-Murcia et al., 2014a; Martinez-Murcia et al., 2016b; Khedher et al., 2015; Pereira et al., 2015; Badoud et al., 2016). Although there are others, such as Naïve-Bayes (Towey et al., 2011) or logistic lasso (Tagare et al., 2017), in line with general trends, SVM, with linear or radial basis function kernel, has been the most commonly employed tool, and in the last years the use of methods based on artificial neural networks (ANN) have gained popularity.

The development of novel architectures and effective training algorithms has enabled to use multi-layer neural networks or deep neural networks (aka deep learning) for a wide range of applications (LeCun et al., 2015), such as speech recognition (Hinton et al., 2012), drug discovery (Chen et al., 2018) and genomics (Alipanahi et al., 2015), but it is in the field of computer vision and image classification where deep learning, and particularly convolutional neural networks (CNN), has undergone a real revolution of the state of the art (LeCun et al., 2015). CNNs are biologically-inspired models that resemble the human vision system, computing image features at different abstraction levels by means of the convolution operator, which is subsequently applied to the response of the previous layer (Rawat and Wang, 2017). Nowadays, these architectures have practically reached, or even surpassed, human-level performance in object recognition (Kheradpisheh et al., 2016). Two of the most famous CNN architectures are LeNet-5 (LeCun et al., 1998) and AlexNet (Krizhevsky et al., 2012). They have been well-studied and provide good results compared to other machine learning algorithms and even more complex CNNs. In fact, deeper networks (e.g., Inception Szegedy et al., 2015), with higher number of abstraction levels, allow computing more complex features, but they also result much more complex to train. This causes that the performances degrade because the limitations of the training algorithms (He et al., 2016) and that the architectures tend to be overffited. Thus, although deeper architectures have the potential to outperform simpler LeNet-5 and AlexNet, this cannot be always achieved and even so, the gain in accuracy may imply a considerable higher computational burden that may not be always justified (Martinez-Murcia et al., 2018).

This work analyzes DaTSCAN (3D) images and identifies features which are suitable for being used in a computer-aided classification system intended to classify between positive and negative cases of PD. In particular, this is realized through the identification of isosurfaces and the extraction of descriptive features from these by using CNN architectures based on LeNet-5 and AlexNet. Isosurfaces connect voxels that have the specified intensity or value, much the way contour lines connect points of equal elevation. This work culminates in the implementation of a classification system which uses supervised learning through CNN architectures to classify DaTSCAN images with an average accuracy of 95.1%. Sensitivity and specificity of the system have also been calculated resulting at an average of 95.5% and 94.8%, respectively.

After this introduction, the rest of the paper is structured as follows. Section 2 reviews related works for PD diagnosis. Section 3 shows details on the database used in this work, extracted from the Parkinson Progression Neuroimaging Initiative (PPMI, RRID:SCR_006431) database, and the applied preprocessing. Then, section 4 describes the computing of isosurfaces, the analyzed architectures and their training process. Section 5 presents and discusses the classification results using data from the PPMI. And finally, section 6 shows the conclusions drawn from this work along with its practical applicability.

## 2. Related Work

The high spatial and color resolution provided by current neuroimaging systems has prompted them to become the main diagnosis tool for neurodegenerative disorders. Thus, DaTSCAN SPECT imaging is used routinely for the diagnosis of PD through the evaluation of deficits of dopamine transporters of the nigrostriatal pathway. However, the visual assessment of these images to come to a final diagnostic is, even for expert clinician, a time-consuming and complicate task, which requires having into account many variables. Machine learning algorithms, which allow combining different types of inputs to produce a result, can potentially overcome this problem. Additionally, the vast amount of information contained in DatSCAN images requires the use of computer aided tools to be exploited, allowing to find complex, disease-related patterns to increase the diagnosis accuracy. We review next the main computer-based techniques proposed in this framework.

Two of the first works to analyze the possibilities of machine learning algorithms with DaTSCAN were Palumbo et al. (2010) and Towey et al. (2011). The former compared a probabilistic neural network (PNN) with a classification tree (CIT) to differentiate between PD and essential tremor. Striatal binding ratios for caudate and putamina on 3 slices were used as image features. The latter used Naïve-Bayes with PCA decomposition of the voxels in the striatal region. These were followed for a series of works where SVMs were used as the main classifier tool, with linear or RBF kernel and different image features. Illán et al. (2012) and later Oliveira and Castelo-Branco (2015) used voxel-as-features; i.e., image voxel intensities are used directly as features. Segovia et al. (2012) used a Partial Least Square (PLS) scheme to decompose DaT images into scores and loading. Then, the scores with the highest Fisher Discriminant Ratios were used as feature for the SVM. Khedher et al. (2015) also used PLS. Rojas et al. (2013) proposed the use of 2D empical mode decomposition to split DaTSCAN images into different intrinsic mode functions, accounting for different frequency subbands. The components were used to select features related to PD that clearly differentiate them from NC, allowing an easy visual inspection. Martínez-Murcia et al. (2014a) decomposed the DaTSCAN images into statistically independent components which revealed patterns associated to PD. Moreover, in this approach, image voxels were ranked by means of their statistical significance in class discrimination. A more recent approach also based on multivariate decomposition techniques is proposed in Ortiz et al. (2018), where the use of functional principal component analysis on 3D images is proposed. This is addressed by sampling the 3D images using fractal curves in order to transform the 3D DatSCAN images into 1D signals, preserving the neighborhood relationship among voxels. Striatal binding ratios for both caudates and putamina were used in Prashanth et al. (2014), Palumbo et al. (2014), and Bhalchandra et al. (2015). Martínez-Murcia et al. (2014b) proposed the extraction of 3D textural-based features (Haralick texture features) for the characterization of the dopamine transporters concentration in the image. And finishing with those based on SVM, Badoud et al. (2016) used univariate (voxel-wise) statistical parametric mapping and multivariate pattern recognition using linear discriminant classifiers to differentiate among different Parkinsonian syndromes.

More recently, methods based on neural networks, especially deep learning-based methods, have paved the way to discover complex patterns and, consequently, to outperform the diagnosis accuracy obtained by classical statistical methodologies (Ortiz et al., 2016; Martinez-Murcia et al., 2017). The use of models containing stacks of layers composed of a large number of units that individually perform simple operations allows to compute models containing a large number of parameters. Moreover, these massively parallelized architectures are able to discover very complex patterns in the data by a learning process formulated as an optimization problem. Zhang and Kagen (2017) proposes a classifier based on a single layer neural network and voxel-as-features from different slices. Martinez-Murcia et al. (2017) and Martinez-Murcia et al. (2018) propose the use of Convolutional Neural Networks (CNN) to discover patterns associated to PD. Increasing the accuracy requires the use of deeper networks, but this increment also makes the network prone to overfitting and push the training algorithms to their performance limits. Thus, architectures combining more elaborated blocks such as in He et al. (2016) have been also proposed to effectively increase the number of layers.

In this work, we describe a classifier based on the well-known CNNs LeNet-5 and AlexNet where the image features used to train them are isosurfaces computed from the regions of interest. The computation of isosurfaces reduces the complexity of the inputs significantly which results in high classification accuracies with reduced computational burden.

## 3. Materials

### 3.1. Database

Data used in the preparation of this article was obtained from the PPMI (Parkinson's Progression Markers Initiative, RRID:SCR_006431). PPMI is an observational clinical study to verify progression markers in PD. For up-to-date information on the study, visit https://www.ppmi-info.org/. The images in this database were imaged 4 + 0.5 h after the injection of between 111 and 185 MBq of DaTSCAN. Raw projection data are acquired into a 128 × 128 matrix stepping each 3 degrees for a total of 120 projection into two 20% symmetric photopeak windows centered on 159 KeV and 122 KeV with a total scan duration of approximately 30–45 min (The Parkinson Progression Markers Initiative, 2010).

A total of *N* = 269 DaTSCAN images from this database were used in the preparation of the article. Specifically, the baseline acquisition from 158 subjects suffering from PD and 111 normal controls (NC) was used.

### 3.2. Spatial Normalization

Spatial normalization is frequently used in neuroimaging studies. It eliminates differences in shape and size of brain, as well as local inhomogeneities due to individual anatomic particularities. It is particularly key in group analysis, where voxel-wise differences are analyzed and quantified (Martinez-Murcia et al., 2016a). In this procedure, individual images are mapped from their individual subject space (image space) to a common reference space, usually stated using a template. The mapping involves the minimization of a cost function that quantifies the differences between the individual image space and the template. The most frequent template is the Montreal Neurological Institute (MNI), set by the International Consortium for Brain Mapping (ICBM) as its standard template, currently in its version ICBM152 (Mazziotta et al., 2001), an average of 152 normal MRI scans in a common space using a nine-parameter linear transformation. A particular case of affine transformation is the similarity transformation, where only scale, translation and rotation are applied. This is often used for motion correction and reorientation of brain images with respect to a reference, and is frequently performed automatically on many imaging equipment. The DaTSCAN images from the PPMI dataset are roughly realigned. We will refer to this as non-normalized (given that it is only a similarity transformation that preserves shape) or “original.” We further preprocessed the images using the SPM12 (Functional Imaging Laboratory of the University College London, 2012) New Normalize procedure with default parameters, which applies affine and local deformations to achieve the best warping of the images and a custom DaTSCAN template defined in Salas-Gonzalez et al. (2015).

Finally, the regions of interests, those which reveal dopaminergic activity, were selected. As a result, the images of original size of (95, 69, 79) were converted into images of size (29, 25, 41). This means passing from 498,800 to 29,795 voxels, a diminution of 94%, which reduces dramatically the complexity of the system without losing almost relevant information since beyond the elected area the intensity values of most of the pixels for both groups is very low or zero.

### 3.3. Intensity Normalization

Intensity normalization is an important step to ensure that the same intensity levels corresponds to similar drug uptakes, so that intensities can be compared as an indirect measure of the neurophysical activity. Similar intensity values should indicate similar drug uptakes and, as a consequence, differences in these values may reveal different pathologies (Martínez-Murcia et al., 2012; Segovia et al., 2012; Padilla et al., 2015).

This paper uses Integral Normalization (Illán et al., 2012):

(1) $$\begin{array}{r} {\hat{\text{I}}}_{i}={\text{I}}_{i}/I_{n,i}, \end{array}$$

where **I**_*i*_ is the image of the *ith* subject in the dataset, ${\hat{\text{I}}}_{i}$ is the normalized image, and *I*_*n*_ is an intensity normalization value that is computed independently for each subject as the mean of the whole image (in an approximation of the integral). Sometimes, for Parkinson studies, *I*_*n*_ is set to the average of the brain without the striatum; although the influence of this is small and it can be approximated by the mean of the whole image. Finally, in this work, the resulting values are further normalized between 0 and 1.

## 4. Methods

### 4.1. Feature Extraction Using Isosurfaces

DaTSCAN SPECT images contains an enormous amount of information. The approach of using voxels-as-features has been adopted by different works (Illán et al., 2012; Badoud et al., 2016) reporting modest classification results around 70–75% and suggesting that better results can be achieved by using more refined techniques that focus on the significant information that lies in such images. When CNN are used, as mentioned in the previous sections, this can be explained by a reduction in the complexity that results in lower computational burden (Martinez-Murcia et al., 2018), more efficient training algorithms (He et al., 2016) and less proneness to overfitting. The extraction and selection of features is therefore one of the most determinant processes, and maybe the most characteristic part, in the definition of a classification method.

For feature extraction, this paper proposes the use of isosurfaces. Isosurfaces connect voxels that have the specified intensity or value much the way contour lines connect points of equal elevation. Roughly, this implies to set a threshold at a certain level and take the surface that envelops the remaining voxels above that threshold. In this work, however, a refined version for computing isosurfaces is used where interpolation is employed instead of just thresholding.

**Figure 1:**
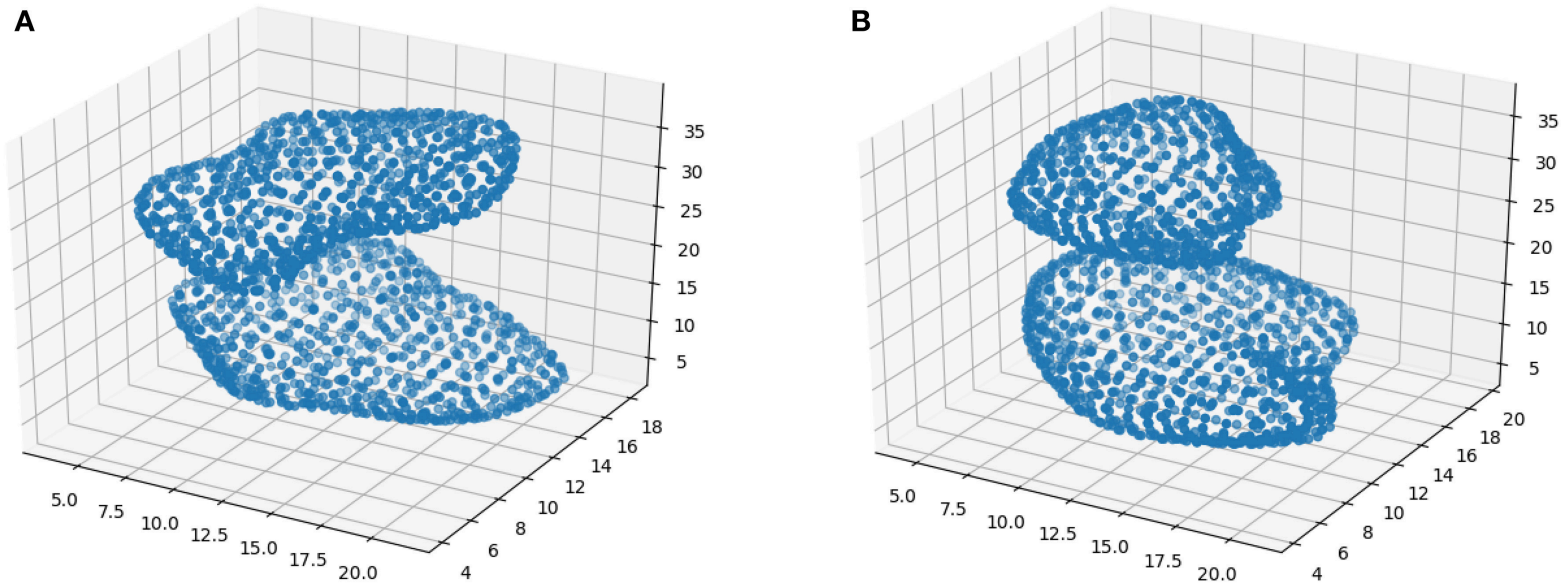
Examples of isosurfaces with threshold = 0.5 for a NC subject **(A)** and PD patient **(B)**.

**Figure 2:**
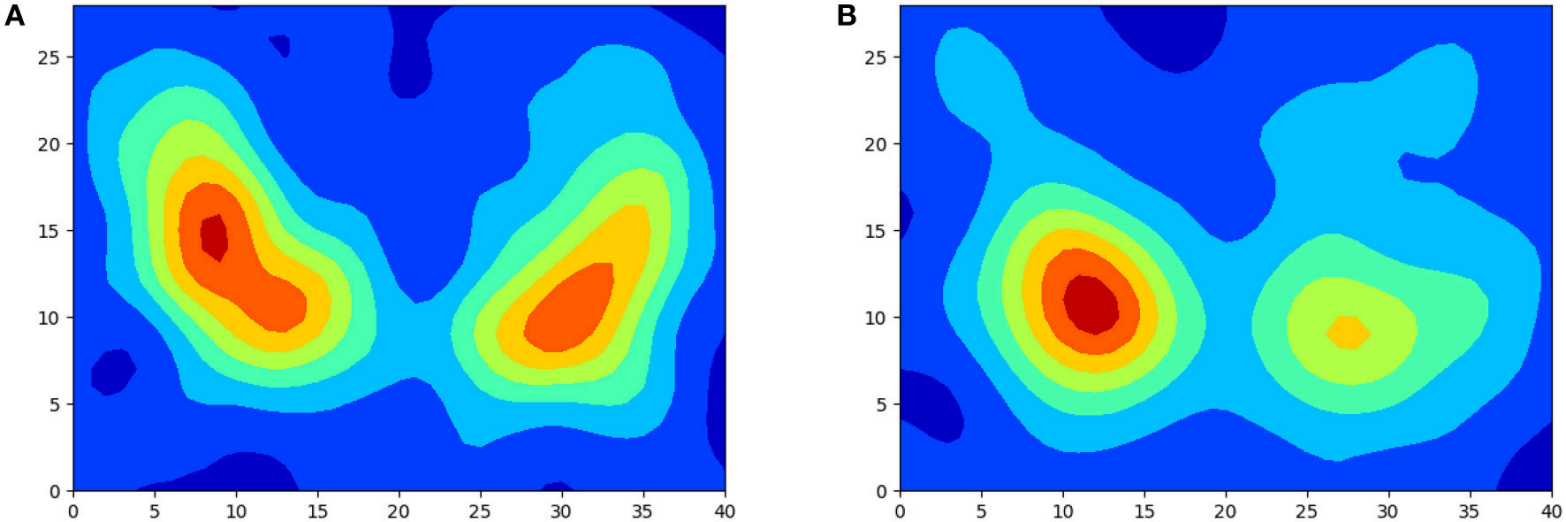
Examples of isolines with different threshold for a NC subject **(A)** and PD patient **(B)**.

Figure 1 shows two examples of isosurfaces computed with a threshold of 0.5 (intensity is normalized to 1) for a NC subject and a PD patient. Unfortunately, it is difficult to observe in a figure different isosurfaces computed for different thresholds since that with the highest threshold will envelop the rest. As an alternative, when different thresholds are used, isolines are preferred. Isolines are simply 2D slices of the corresponding isosurfaces. In Figure 2 isolines with different thesholds for a NC subject and a PD patient are represented. The following characteristics can be observed in isosurfaces/isolines: (i) they define closed volumes/areas, (ii) they do not cross each other, (iii) the same threshold can result in several isosurfaces/isolines, and (iv) the proximity between isosurfaces/isolines provides information about intensity gradients; the closer they are, the faster the changes. Regarding the diagnosis of PD, it can be observed in previous figures that isosurfaces and isolines from PD patients, in contrast with those from NC subjects, are characterized by a loss of symmetry between hemispheres.

Feature selection is usually based on either statistical analysis or optimization of the classifier. In the former, previously computed thresholds based on statistical relevance or correlations are set and features are discarded if they are not above such thresholds without considering the performance of the classifier. In the latter, however, features are selected, or discarded, if they improve, or not, the performance of the classifier. This paper uses this second approach. Classification results using isosurfaces computed with different thresholds have been compared, choosing those that provide the best classification results. More specifically, isosurfaces for thresholds 0.4, 0.5, 0.6, 0.7, and 0.8 have been computed. Then, the two possible options have been analyzed: forward selection, where just one isosurface for a threshold is initially used with the classifier and then others are gradually included if they improve the results; and backward selection, where the whole set of isosurfaces is initially employed to classify and then some of these are removed if their absence does not affect negatively the classification performance.

### 4.2. CNNs for Classification

Method based on neural networks are becoming more and more popular for the development of new early diagnosis tools (Ortiz et al., 2016). More specifically, CNNs have been proposed for the detection of patterns in medical images associated to PD (Martinez-Murcia et al., 2017, 2018). The election and configuration of the CNN architecture are, however, not trivial tasks. In fact, although deeper structures, with higher numbers of layer and units, are potentially more capable of revealing hidden patterns, they are not always advisable because the complexity that they introduce. When a big amount of parameters need to be adjusted, it may result in training problems, overfitting and high computational loads. Thus, apart from preprocessing input data to remove non-significant information and feed CNNs with relevant inputs, the best performances are obtained with balanced architectures; that is, architectures complex enough to reveal the relevant patterns but not so complex that it cannot be conveniently trained with certain guarantees of non-overfitting. In this paper, two 3D versions based on well-known architectures have been tested. The first based on LeNet (LeCun et al., 1998), and then another based on the most powerful AlexNet (Krizhevsky et al., 2012), both of them fed with pre-processed data resulting from the computation of the isosurfaces.

**Figure 3:**
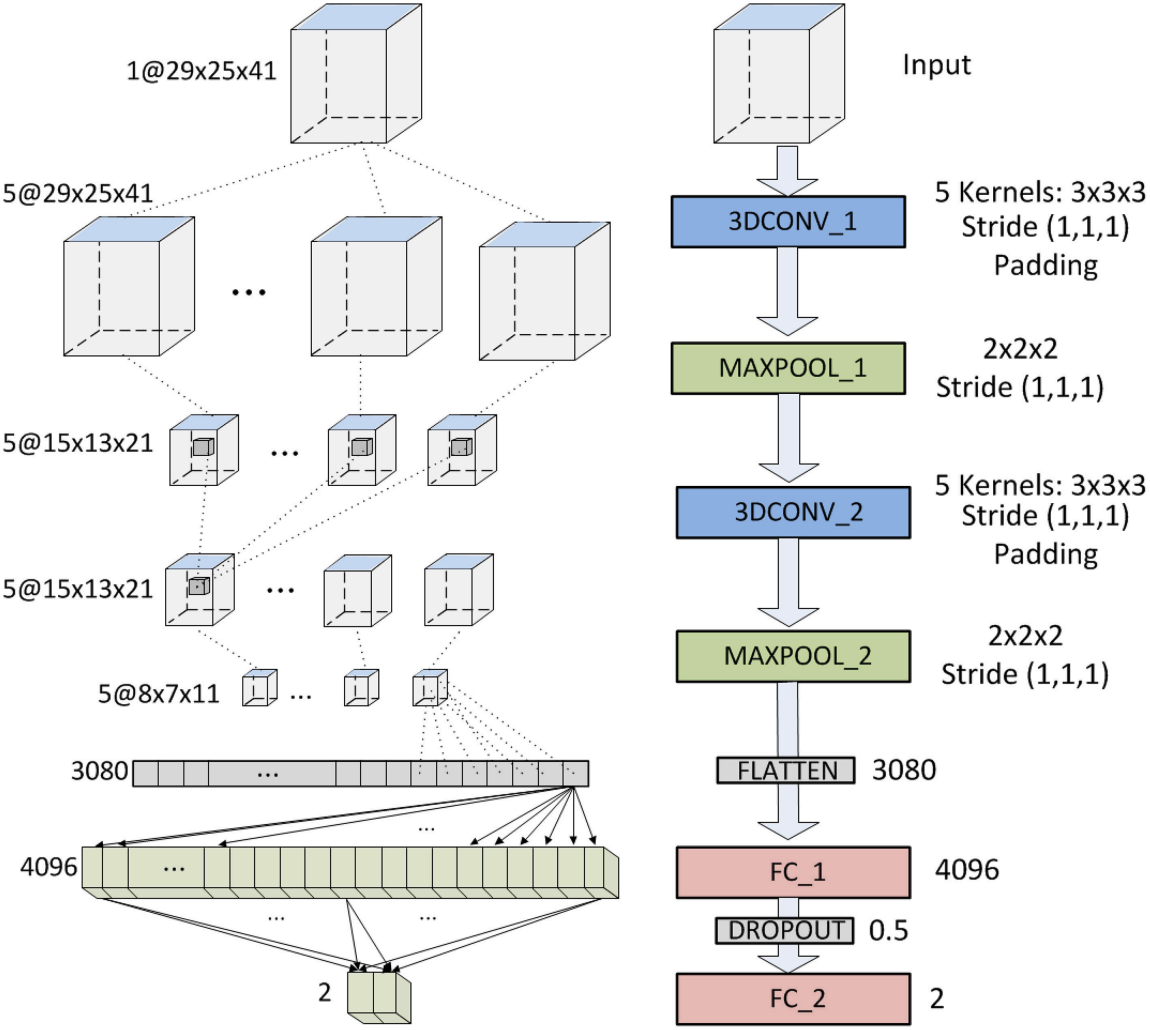
CNN architecture based on LeNet.

Our first architecture comprises 7 layers, not counting the input (see Figure 3) : 2 convolutional layers (first and third), 2 subsampling layers (second and fourth), 1 flatten layer (fith) and 2 full connected layers (sixth and seventh). The 2 convolutional layers use five 3D-kernels of [3 × 3 × 3] to sweep over the input topologies and transform them into feature maps. Stride of (1,1,1) and padding are employed with the convolution so that the output feature maps keep the size of the input. For the second convolutional layer (3DCONV_2), each unit in each feature map is connected to [3 × 3 × 3] neighborhoods at identical locations in the entire set of input feature maps. Thus, the number of trainable parameters of these two layers are 3^3^ * 5 + 5 = 140 and 3^3^ * 5 * 5 + 5 = 680, respectively. Note however, that the number of trainable parameters of the first layer increases if several images are introduces simultaneously (*#params* = 3^3^ * 5 * *#inputs* + 5). The two subsampling layers apply max-pooling, connecting each unit in the output feature map to [2 × 2 × 2] neighborhood in the input feature map. The output is the maximum within the [2 × 2 × 2] window. Consequently, the output feature maps have half the number of units in the three dimensions. Sub-sampling reduces the complexity of the CNN and provides invariance to local translations. Once the feature learning phase is completed, using the convolutional and sub-sampling layers, feature maps are flattened into a feature vector. This vector consists of 8 * 7 * 11 * 5 = 3, 080 neurons, and is followed by two fully-connected layers of 4,096 and 2 neurons, respectively. The number of trainable parameters of the last two layers are 3, 080 * 4, 096 + 4, 096 = 12, 6190, 776 and 4, 096 * 2 + 2 = 8, 194, respectively. Between these two layers there is a dropout interphase with 0.5 dropout probability. The last layer yields the prediction probability using softmax activation. The total number of trainable parameters of this CNN is 12,628,790.

**Figure 4:**
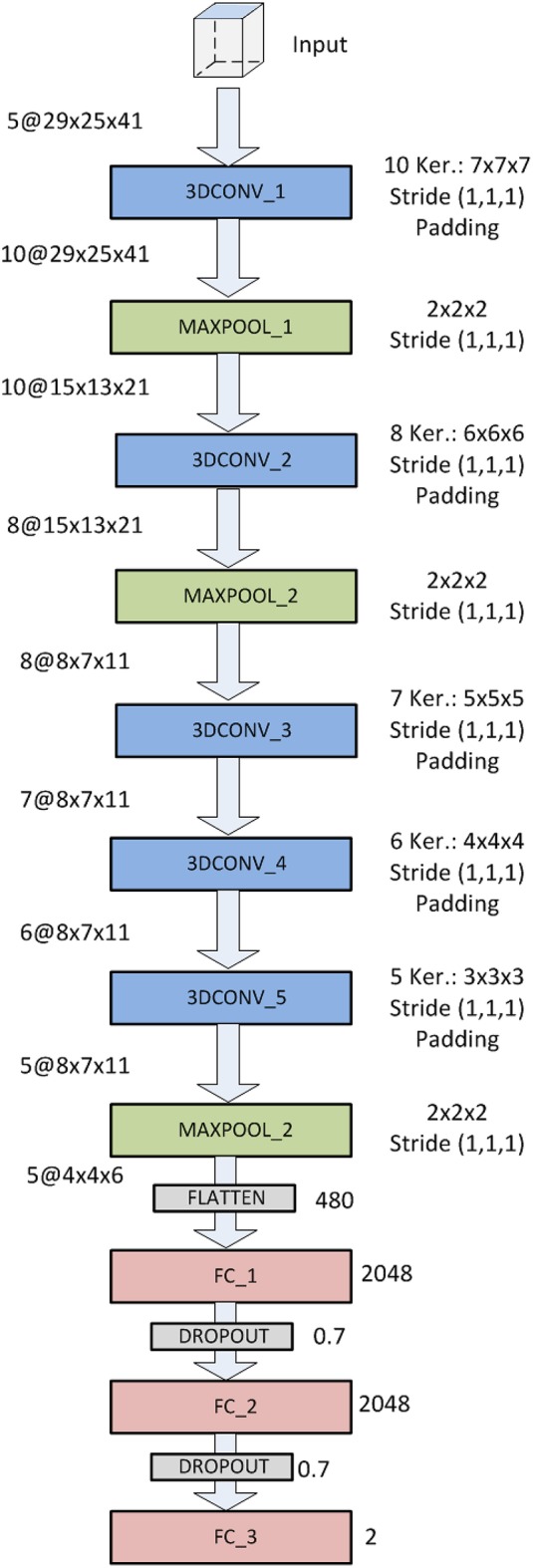
CNN architecture based on AlexNet.

The AlexNet based architecture is shown in Figure 4. It comprises 12 layers: 5 (first, third, fourth, fifth and sixth) 3D-convolutional layers, 3 (second, fourth and eight) max-pooling (subsampling) layers, 1 flatten layer (ninth) and 3 fully-connected layers (tenth, eleventh and twelfth). The convolutional layers use 10, 8, 7, 6 and 5 kernels of sizes [7 × 7 × 7], [6 × 6 × 6], [5 × 5 × 5], [4 × 4 × 4] and [3 × 3 × 3], respectively. Convolutional layers use padding and stride (1,1,1), and output feature maps are connected to every input feature map (not just a subset). The flatten layer has 480 neurons and the three last fully connected layers 2,048, 2,048, and 2, respectively. Between these three fully connected layers there are two dropout interphases with dropout probability of 0.7. The last two-neuron layers uses softmax activation to predict a classification. These characteristics and information about the number of trainable parameters of this CNN are summarized in Table 1.

**Table 1:** Characteristics of the AlexNet-based CNN used.

**Layer**	**Kernel/Window**	**Output shape**	**Trainable parameters**
Input		29 × 25 × 41	
3D-Conv_1	10@7 × 7 × 7	10@29 × 25 × 41	3,440^*^
Max_pool_1	2 × 2 × 2	10@15 × 13 × 21	0
3D-Conv_2	8@6 × 6 × 6	8@15 × 13 × 21	17,288
Max_pool_2	2 × 2 × 2	8@8 × 7 × 11	0
3D-Conv_3	7@5 × 5 × 5	7@8 × 7 × 11	7,007
3D-Conv_4	6@4 × 4 × 4	6@8 × 7 × 11	2,694
3D-Conv_5	5@3 × 3 × 3	5@8 × 7 × 11	815
Max_pool_3	2 × 2 × 2	5@4 × 4 × 6	0
Flatten		480	0
FC_1		2,048	985,088
FC_2		2,048	4,196,352
FC_3		2	4,098
		Total	5,216,782

* *Computed by a single input volume*.

### 4.3. Evaluation

Classification performance is evaluated by means of the accuracy, sensitivity and specificity. Resulting from these values, Receiver Operating Curves (ROC) and the Areas Under the ROC Curves are also computed. ROC curves comprise the sensitivity and specificity to provide compromise values between these two values, while AUC provides a metric regarding the performance of the classifier.

Classification experiments conducted in this work have been assessed by nested cross-validation (Stone, 1974), with inner and outer loops implementing stratified k-fold cross-validation (*k*=10) to ensure that the proportion of both classes is preserved in each fold. The inner loop is used to select the features and the outer to determine the generalization ability of the proposed method (Lozano et al., 2017). However, in order to provide a sweep of the performances obtained for different thresholds and to carry out a fair comparison with the optimal one, the results of the outer loop are provided for the different used values (even when they were not the optimal in the inner loop). Estimation of the generalization error by cross-validation will always result in an overestimate in practice, since the entire training set is not used but just a fraction. This overestimate will depend on the slope of the learning curve of the classifier and reduces when *k* increases.

Standard error is computed from the standard deviation. Cross-validations performed for *k* << *N* (where *N* is the number of samples) allow to estimate the standard deviation of an experiment *CV*(ζ). First, the validation error in the *j*-th fold is averaged as

(2) $$\begin{array}{r} CV_{j}\left(\zeta \right)=\frac{1}{n_{j}}e_{j}\left(\zeta \right)=\frac{1}{n_{j}}\underset{i\in F_{j}}{\sum }{\left(y_{i}-{\hat{f}}_{\zeta }^{-j}\left(x_{i}\right)\right)}^{2} \end{array}$$

where *n*_*j*_ is the number of samples in the *j*-th fold. Then, the standard deviation of *CV*_*j*_(ζ) with 1 ≤ *j* ≤ *k* can be computed as:

(3) $$\begin{array}{r} SD\left(\zeta \right)=\sqrt{var\left(CV_{1}\left(\zeta \right),\ldots ,CV_{k}\left(\zeta \right)\right)} \end{array}$$

where *var*(*x*) stands for the variance of the vector *x*. Finally, the standard error [or standard deviation of *CV*(ζ)] is computed as:

(4) $$\begin{array}{r} SEM\left(\zeta \right)=k^{-\frac{1}{2}}SD\left(\zeta \right) \end{array}$$

## 5. Results and Discussion

In this section, we firstly compare classification results when just a single input volume (isosurface) is introduced in the LeNet-based and AlexNet-based architectures. This allows determining which isosurfaces provide more significant information and comparing the performances of both architectures.

**Figure 5:**
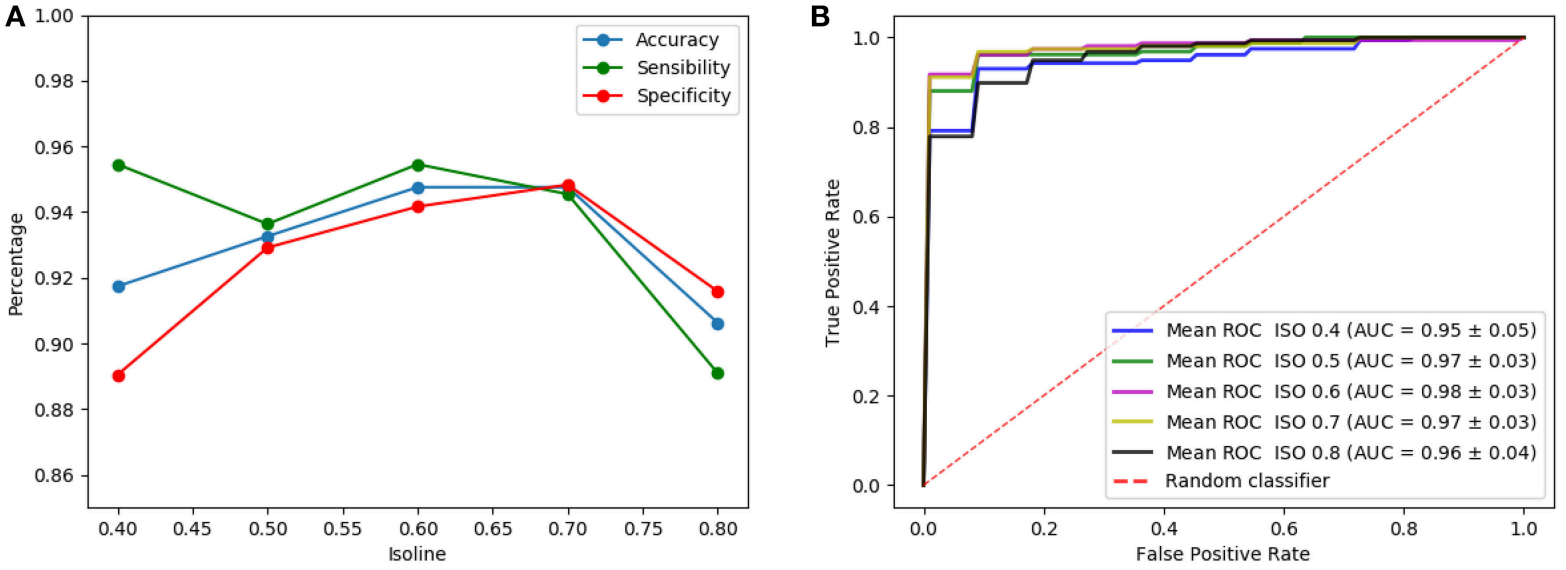
Results of the LeNet-based architecture using as input a single isosurface: sensibilities, sensitivities and accuracies **(A)** and ROC curves **(B)**.

**Figure 6:**
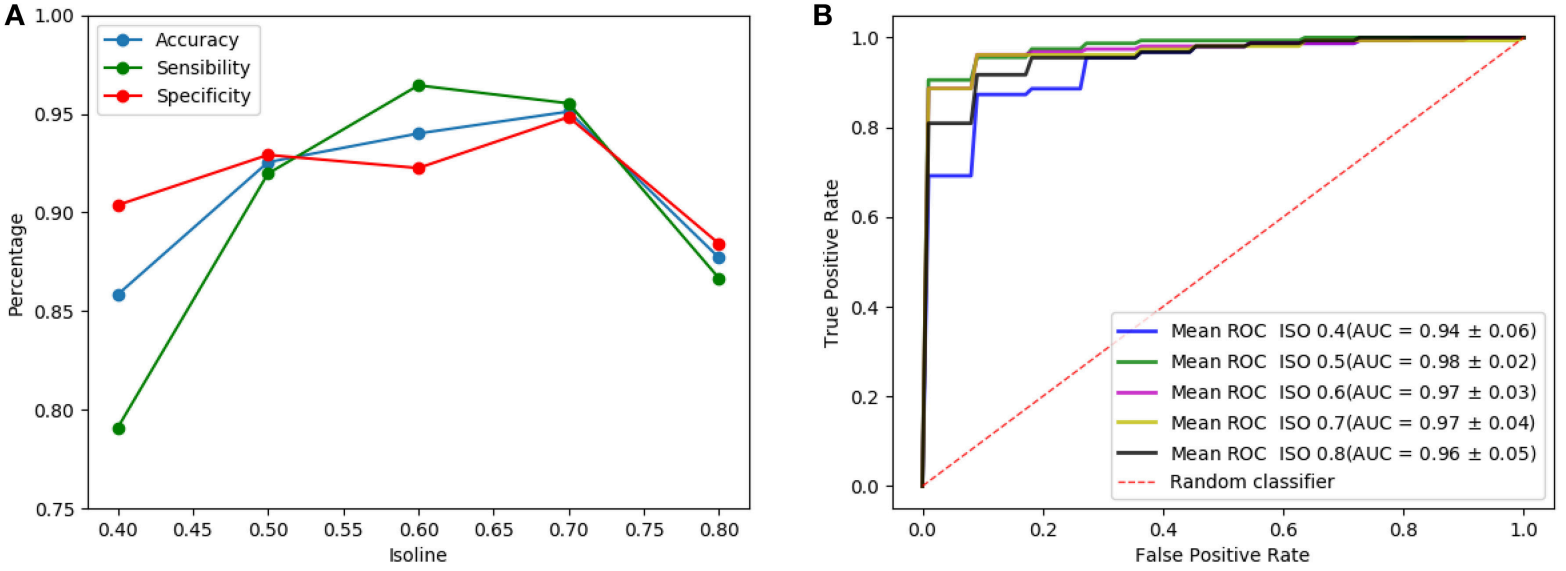
Results of the AlexNet-based architecture using as input a single isosurface: sensibilities, sensitivities and accuracies **(A)** and ROC curves **(B)**.

Figure 5 shows the results of the LeNet-based architecture for the computed isosurfaces (see section 4.1); Figure 5A graphs sensibilities, sensitivities and accuracies, and Figure 5B the ROC curves. Likewise, Figure 6 shows the results for the AlexNet-based architecture. Classification performances increase slightly with the threshold, until this is 0.7. This is explained because the greater the threshold the less the volume captured by the isosurfaces. Thus, as the threshold increases but the chosen volume still contains most of the relevant regions (around the striatum) the performances maintain or improve, since the computational complexity reduces while keeping the significant information. However, for thresholds beyond 0.8, the captured area reduces too much, leaving out relevant regions for the classification and therefore decreasing the performances. As a result, intermediate values of isosurfaces, i.e., 0.5, 0.6, and 0.7, seem to contain the most relevant information providing slightly better classification results for both architectures. On the other hand, there is not a clear difference between the results of the two architectures, both achieving similar performances.

**Figure 7:**
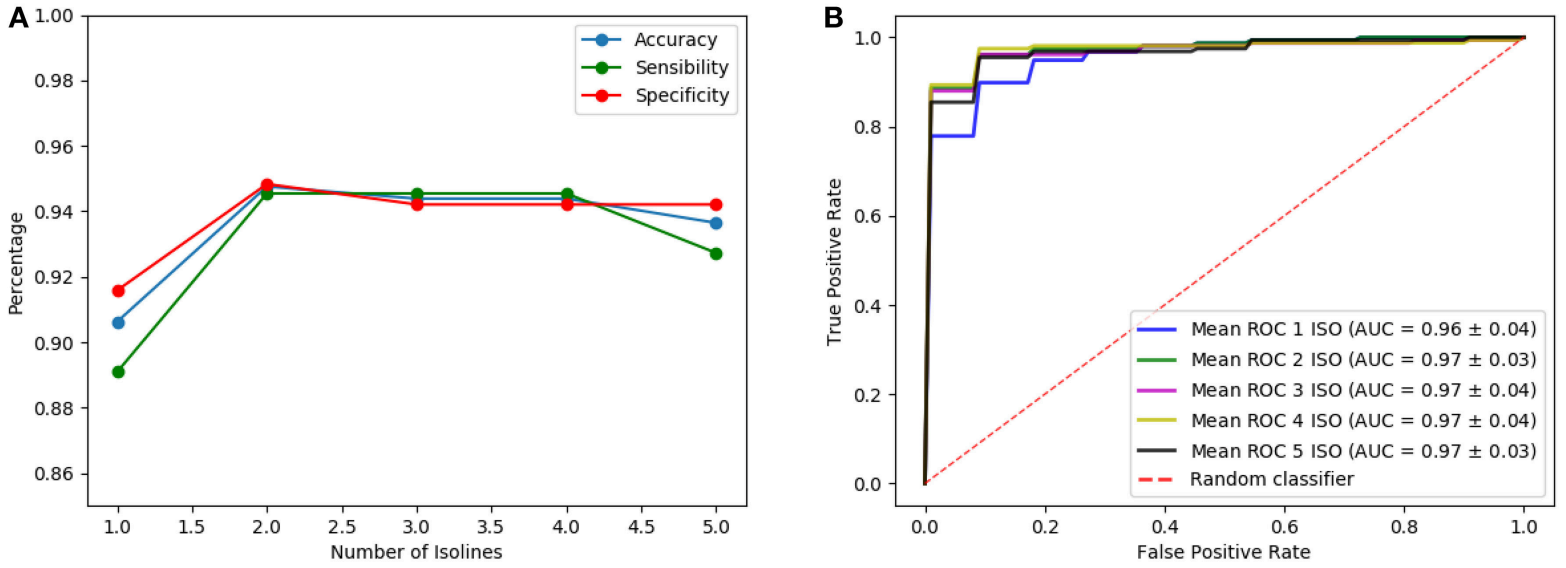
Results of the LeNet-based architecture using as input several isosurfaces: sensibilities, sensitivities and accuracies **(A)** and ROC curves **(B)**.

**Figure 8:**
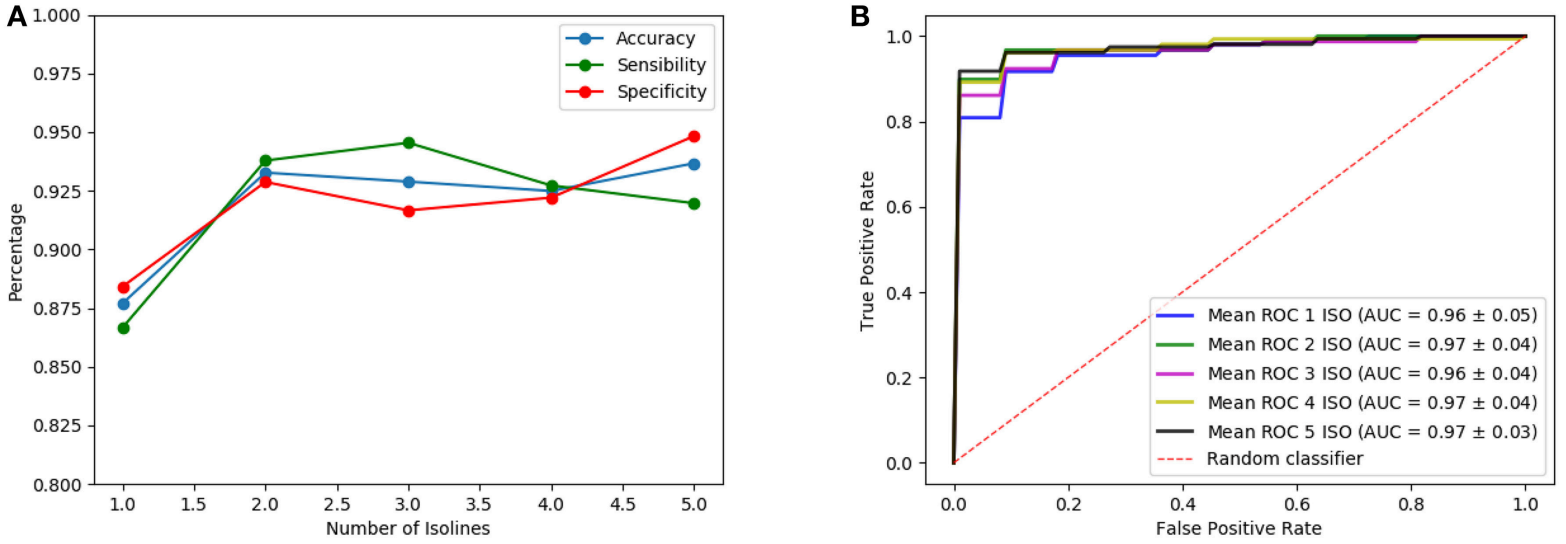
Results of the AlexNet-based architecture using as input several isosurfaces: sensibilities, sensitivities and accuracies **(A)** and ROC curves **(B)**.

Once the analysis using isosurfaces independently is completed, classification performances obtained when different number of isosurfaces are used as input of the architectures are compared. Note that, although the introduction of more isosurfaces adds more information, it also increases the complexity of the CNN (number of trainable parameters of the first layer) so that the best results are only obtained when the input has an optimum trade-off between the information it provides and the complexity that it introduces. Thus, many possible combinations of isosurfaces have been tested. One of these tests is shown in Figures 7, 8 for the LeNet-based and AlexNet-based architecture, respectively. They show the results for the case where isosurfaces are sequentially added from top level (0.8) to bottom level (0.4); that is, first the isosurface with level 0.8 is used by itself (marked as 1 in the figures), then 0.7 is added (2 in the figures), next 0.6 is also added (3 in the figures) and so on: 0.8+0.7+0.6+0.5 (4 in the figures) and all of them (5 in the figures).

The inputs chosen eventually as providing the best classification results while keeping the complexity as low as possible have been the combination of isosurfaces 0.8 and 0.7 for the LeNet-based architecture and the isosurface 0.7 for the AlexNet-based architecture. They both provide accuracy, sensibility and specificity about 0.95 and AUC = 0.97. These classification performances can be considered as very good when compared with other well-known methods such as VAF (Voxels as Features), PCA (Principal Component Analysis) or EfPCA (Empirical functional PCA), outperforming most methods recently published in the bibliography for the detection of Parkinsonism (Rojas et al., 2013; Martínez-Murcia et al., 2014a; Brahim et al., 2015). Table 2 collects the different performance classifications, including the typical deviation when available. Additionally, in order to statistically confirm the effectiveness of the proposed method, a statistical hypothesis test (Welch test) has been performed in terms of the AUC. As a result, the statistical significance of the use of isosurfaces along with the LeNet-based architecture when compared with the EfPCA (the next best performing method in the comparison) is confirmed with a *p*-value of 0.04. By contrast, a *p*-value of 0.18 is computed when both architectures, LeNet and AleNet based, are compared, which allows to infer that while the use of isosurfaces as a feature extraction method outperforms previous approaches, it is not possible to state if one of the two architectures performs better than the other.

**Table 2:** Classification results using different methods.

**Method**	**Accuracy**	**Sensitivity**	**Specificity**	**AUC**
EMD (Rojas et al., 2013)	0.95	0.95	0.94	0.94
Significance M. (Martínez-Murcia et al., 2014a)	0.92	0.95	0.89	0.90
Brahim et al. (2015)	0.92	0.94	0.91	–
VAF	0.8 ± 0.05	0.72 ± 0.17	0.85 ± 0.14	0.87
PCA	0.87 ± 0.04	0.96 ± 0.03	0.86 ± 0.04	0.9
EfPCA (Ortiz et al., 2018)	0.93 ± 0.05	0.97 ± 0.08	0.88 ± 0.05	0.94
**LeNet-based**	**0.95** ± **0.03**	**0.94** ± **0.04**	**0.95** ± **0.04**	**0.97**
**AlexNet-based**	**0.95** ± **0.03**	**0.95** ± **0.05**	**0.95** ± **0.04**	**0.97**

**Figure 9:**
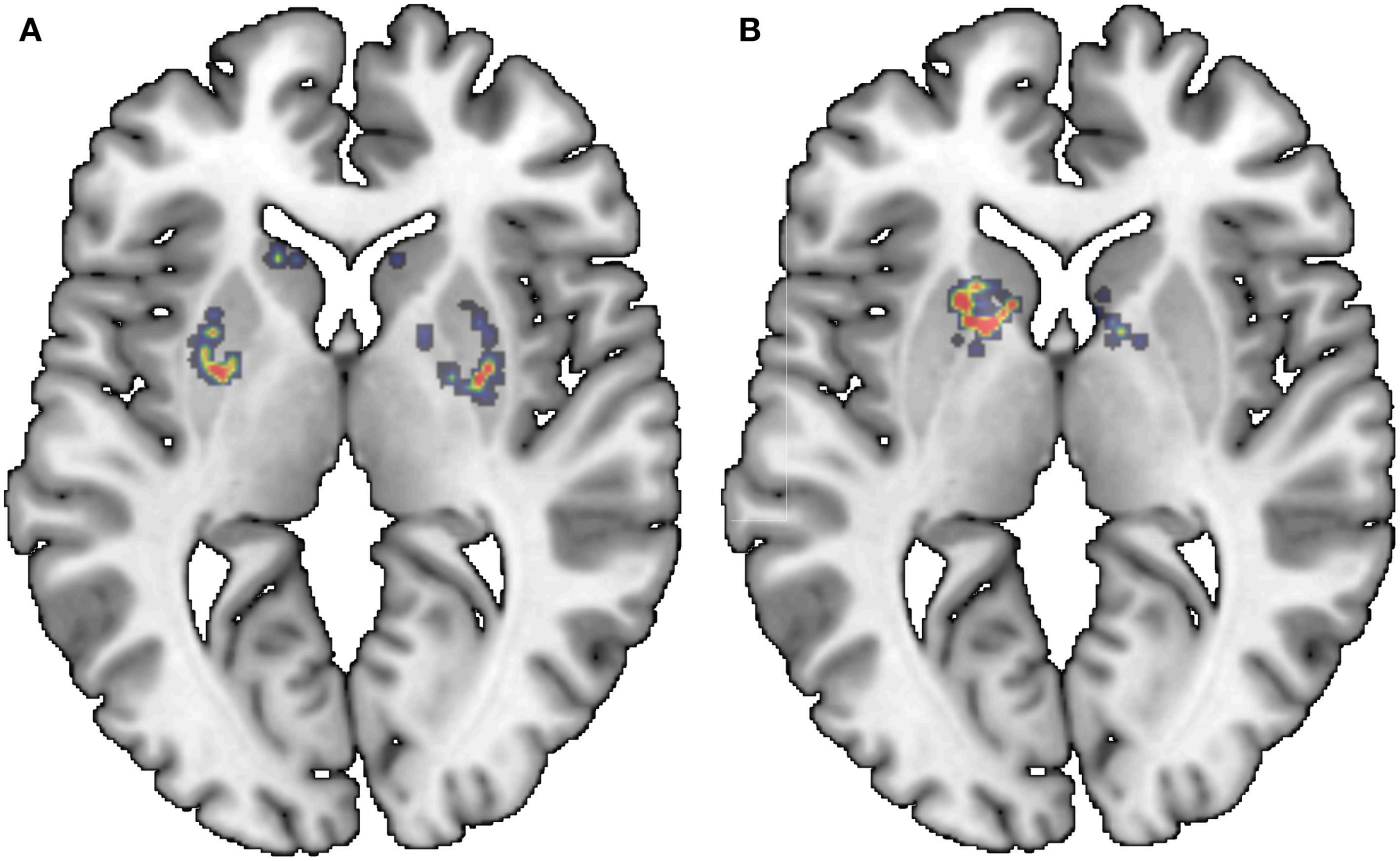
Saliency maps of the LeNet-based architecture superimposed on a MRI image: NC **(A)** and PD **(B)**.

**Figure 10:**
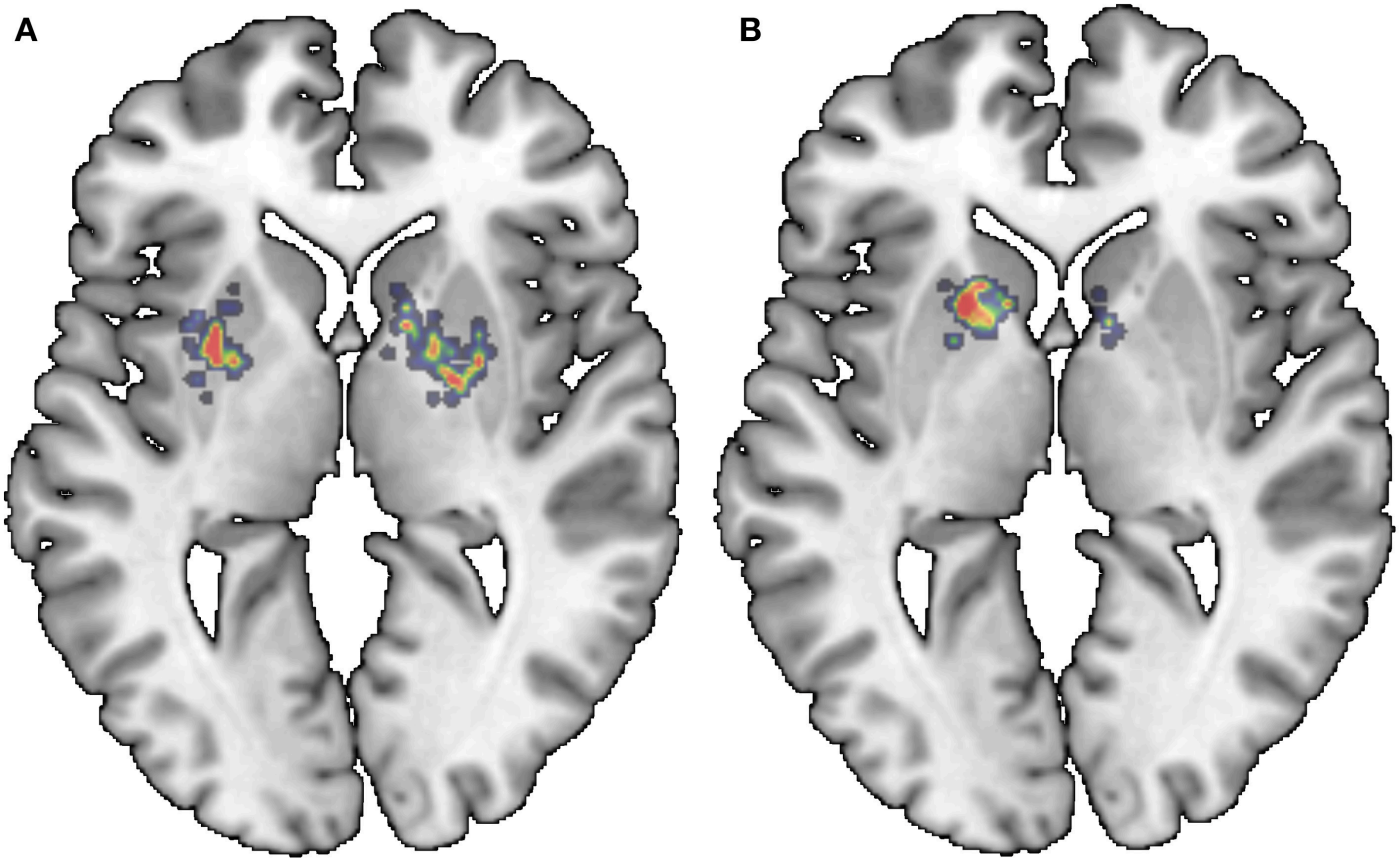
Saliency maps of the AlexNet-based architecture superimposed on a MRI image: NC **(A)** and PD **(B)**.

Finally, and for the sake of completeness, the saliency maps for the last layer of the Lenet-based and AlexNet-based architecture are provided. Figures 9, 10 show a relevant slice of the saliency maps obtained for both architectures superimposed on a MRI image. Saliency maps use the gradient of output category with respect to input image to determine the regions of the input image that have a greater impact on the output class. Thus, for the Alexnet-based architecture (Figure 10), it is observed that for control subjects the most decisive regions are those between the putamen and globus pallidus, while for PD patients, the most important ones are those in the interface between the caudate nucleus and the putamen. Similar regions are found in the case of the LeNet-based classifier. However, in this latter case, for the control subjects, sparser regions are marked in the figure (Figure 9), while for PD patients, it again shows as the most determinant regions the interface between the caudate nucleus and the left putamen. These anatomical regions matched with those reported in the literature (Greenberg et al., 2012; Tuite et al., 2013) as linked to the development of the Parkinson's disease, which confirms the use of isosurfaces as an effective means to extract the most relevant information for PD diagnosis.

## 6. Conclusions

This paper proposes the use of isosurfaces as a way to extract the relevant information from 3D DatSCAN images so that they can be used as inputs of CNN architectures. As a result, a classification system that uses LeNet-based and AlexNet CNN architectures has been implemented. This system achieves accuracy of 95.1% and AUC = 97%, providing comparable (slightly better) values to those obtained for recently proposed systems. It can be concluded, therefore, that the computation of isosurfaces reduces the complexity of the inputs significantly while keeping the relevant information, resulting in high classification accuracies with reduced computational burden. Finally, in order to determine which areas of the input brain images has a greater impact on the predicted output class, saliency maps of the last two-neuron layer are also computed.

## Data Availability

Publicly available datasets were analyzed in this study. This data can be found here: “https://www.ppmi-info.org/” (accessed June 18, 2019).

## Author Contributions

All the authors have been involved in the different phases of the development of this work without being possible to set a clear distinction between the different tasks.

### Conflict of Interest Statement

The authors declare that the research was conducted in the absence of any commercial or financial relationships that could be construed as a potential conflict of interest.
